# In sickness and health - a questionnaire based study regarding immune mediated diseases and neoplasia in Swedish Nova Scotia Duck Tolling Retrievers

**DOI:** 10.1186/s13028-024-00761-x

**Published:** 2024-08-15

**Authors:** Malin Nilsson, Sergey V. Kozyrev, Kerstin Lindblad-Toh, Henrik Rönnberg, Helene Hansson-Hamlin

**Affiliations:** 1 Department of Clinical Sciences, Swedish University of Agricultural Sciences, Uppsala, Sweden; 2 Department of Medical Biochemistry and Microbiology, Uppsala University, Uppsala, Sweden; 3 SciLifeLab, Uppsala University, Uppsala, Sweden; 4 Broad Institute of MIT and Harvard, Cambridge, MA USA

**Keywords:** Autoimmune, Canine tumors, Immune-mediated rheumatic disease, IMRD, Lifetime prevalence, NSDTR, Steroid-responsive meningitis-arteritis, SRMA, Survey

## Abstract

**Background:**

The Nova Scotia Duck Tolling Retriever (NSDTR) has previously been highlighted as a breed at risk for developing immune mediated diseases and cancer. The immune response is of great importance for the development of neoplastic disease and a dysregulated immune response may predispose to cancer. Two of the commonly seen immune mediated diseases in NSDTRs are immune mediated rheumatic disease (IMRD), which bears similarities to systemic lupus erythematosus (SLE) affecting humans, and steroid-responsive meningitis-arteritis (SRMA), which is a non-infectious inflammation of the meninges and the leptomeningeal vessels. The aim of this survey study was to investigate the lifetime prevalence of immune mediated diseases and tumors among Swedish NSDTRs based on owners’ information. The study design was cross-sectional. A questionnaire was sent to 4102 persons who owned or had previously owned a NSDTR. The questions concerned information about the dog and its overall health status as well as specific diseases.

**Results:**

The response rate was 30%, including 935 live NSDTRs, corresponding to approximately 20% of the current population registered in Sweden (*n* = 4564), and 177 dead dogs. The surveyed dogs were spread over different ages and sex and corresponded to the typical demographic profile of the general dog population. Of the 935 individuals that were alive, 28 dogs (3%) were reported as previously diagnosed with IMRD and 33 dogs (3.5%) were reported as previously diagnosed with SRMA, one dog was reported to have been diagnosed with both SRMA and IMRD. There were 129 dogs (14%) reported to have or have had a neoplasia of some kind. For the dead dogs (*n* = 177), almost 40% of the owners reported neoplasia as the main reason for death/euthanasia.

**Conclusion:**

This study reports an estimated lifetime prevalence of IMRD and SRMA, in the studied population of Swedish NSDTRs, of 3.0 and 3.5% respectively. In this study, 14% of the living dogs (*n* = 935) were reported to have a neoplasia of some kind and almost 40% of the deceased dogs (*n* = 177) were euthanized due to neoplasia or suspicion of it.

**Supplementary Information:**

The online version contains supplementary material available at 10.1186/s13028-024-00761-x.

## Background

The first Nova Scotia Duck Tolling Retrievers (NSDTR) came to Sweden in the early 1980’s. Since then the population has increased and there are currently about 4600 NSDTRs registered in Sweden [1]. The NSDTR is a breed loved for its positive attitude and willingness to work. Although healthy in general, studies have shown that immune mediated disease and certain forms of cancer affect NSDTR dogs more often than other breeds [2–5]. Immune mediated rheumatic disease (IMRD) and steroid-responsive meningitis-arteritis (SRMA) are two of the immune-mediated diseases found most commonly in the breed [2–5]. IMRD most often affects middle-aged dogs [3]. It is a chronic condition causing a non-erosive arthritis with affected dogs suffering from stiff gait and pain from several joints and potentially fever, skin ulcers or muscle pain upon palpation [3]. The signs are commonly waxing and waning. The disease is often called SLE-related since it resembles the autoimmune disease systemic lupus erythematosus (SLE) affecting humans [4]. NSDTRs affected with IMRD are generally positive for antinuclear antibodies (ANA), as are SLE patients [4, 6]. Treatment of choice for dogs with IMRD are immunosuppressive doses of glucocorticoids and in most cases, affected dogs respond well to glucocorticoid treatment but sometimes adjuvant treatment, such as azathioprine or cyclosporin A, is needed [4, 7]. Evidence for the best combination of immunomodulatory drugs in these cases is missing and choice of treatment is usually based on the clinicians’ experience and preference [8, 9].

SRMA is a non-infectious meningitis typically causing acute signs of cervical pain, rigidity and pyrexia [5, 10]. It mainly affects young dogs, aged 0.5 to 1.5 years [5, 11]. Treatment of choice involves immunosuppressive doses of glucocorticoids and in general, clinical signs resolve rapidly after treatment is initiated [11, 12]. For cases refractory to treatment with immunosuppressive doses of glucocorticoids, consensus on optimal add-on treatment is missing, but azathioprine is the most widely used secondary treatment in combination with glucocorticoids [13].

A previous study based on insurance data showed that NSDTRs not only have an increased incidence for SRMA and IMRD but also a significantly higher incidence of lymphoma compared to other retrievers as well as all other breeds [3].

The objective of this study was to investigate and potentially estimate the lifetime prevalence of IMRD, SRMA and neoplasia within the breed.

## Materials and methods

### Sampling frame

The target group consisted of all dog owners in Sweden having an NSDTR or that had previously owned a NSDTR. The sampling frame was dog owners registered as owning a NSDTR and having an email address registered at the Swedish Kennel Club (SKK) or the Swedish Board of Agriculture (SJV). A questionnaire-based survey was designed using the web platform Netigate (Netigate AB, Stockholm, Sweden). The questionnaire was adapted for use on personal computers, tablets and smartphones and tested prior to the study by circulation among peers, both animal health care professionals and animal owners.

### Questionnaire and data collection

The final questionnaire was distributed by email and by a link available on the NSDTR Breed Club web page. Email addresses were received from the Swedish Board of Agriculture (18 January 2022) where the request was to access email addresses for all persons registered as owner of a NSDTR. Email addresses were also received from the Swedish Kennel Club (31 January 2022) and the request was to have email addresses to owners with one or more registered NSDTR with a birthdate between 31 January 2007 and 31 January 2022. Overall, that yielded 4102 email addresses. An invitation describing the aim of the study, the general data protection regulation (GDPR) and a link to the questionnaire was sent to all email addresses. Information and survey questions were given in Swedish.

Each owner was asked to answer one questionnaire per dog. The dog did not have to be alive at the time of the survey. If the owner, previously or at the time of answering the survey, owned more than one NSDTR, he or she was invited to answer the questionnaire once for each individual dog.

Data collection was started on 14 March 2022 and was completed on 30 June 2022. Two reminders were sent to non-responders, the first on 14 April 2022 and the second on 20 May 2022.

The questionnaire consisted of 139 questions in total, but depending on the owner’s response to the questions, every owner answered at least 43 questions. No one answered all 139 questions. The questions concerned the animal’s birthdate, sex, neutering or castration status and temper as well as details about diseases affecting various organ systems. They covered aspects related to diagnoses, clinical signs, diagnostic work up and the subsequent treatment, including the response to the prescribed interventions. Questions were either closed, with fixed options for response or open where owners could describe details in own words. The data collected were both nominal and ordinal. Care was taken to make sure that questions were easy to understand as a layman. Many of the diagnoses asked for were referred to both with their name in Latin or Greek but also in Swedish. Regarding neoplasia, the respondents were asked to answer “yes” to the question if the dog had been diagnosed with a neoplasia, regardless of the type of neoplasia, benign as well as malignant or unknown. Owners of dead dogs were asked to report the reason for death/euthanasia. The answers were provided in response to an open question. When several causes were stated as reason for death by the owner, the cause considered to be the most likely one was picked by the researchers. Open questions were categorized to allow for descriptive statistics. A thorough review of the data was performed and incomplete or multiple questionnaires were removed leaving only one complete questionnaire per dog. In case of more than one completed questionnaire for the same dog and from the same respondent, the questionnaire completed first was kept. If two different respondents answered the questionnaire for the same dog, only one answer was kept.

Corrections of answers were made in a few cases where the answer was clearly wrong and the correct answer could easily be found, this was the case for date of birth for a few dogs. Since the owners reported the dog’s registration number, the date of birth could be corrected when wrongly stated, using data from the SKK.

The survey was not anonymous and dog owners were asked to leave their e-mail address and phone number at the end of the survey, although it was possible to complete the questionnaire without leaving contact information.

The complete survey is available as appendix.

### Statistical analysis

Data was analyzed using the statistical software R v4.1.1 (R Foundation for Statistical Computing, Vienna, Austria), with the package “Rcmdr”. Data is presented as median with inter quartile ranges (IQR) since data was not normally distributed. Normality was tested with a Shapiro-Wilk test. For statistical analysis of differences between sexes, one proportion test was used and the significance level was set at 0.05.

## Results

### Response rate

Emails were sent to 4102 addresses and reached 3753 of those. The total number of respondents was 1231, which corresponds to a response rate of 30%. Several respondents answered the survey multiple times but for different individual dogs.

The total number of individual dogs in the survey were 1112 (alive *n* = 935, dead *n* = 177).

### Live dogs

In total we had 935 live dogs in the survey, which corresponds to 20.5% of all registered NSDTRs in Sweden (*n* = 4564) (Jordbruksverket (The Swedish Board of Agriculture), number of dogs per breed from the date 30/12/2021). Of these 935 dogs, 446 (48%) were males and 489 (52%) were females. 127/446 males were castrated (28%) and 74/489 females were neutered (15%).

**Fig. 1:**
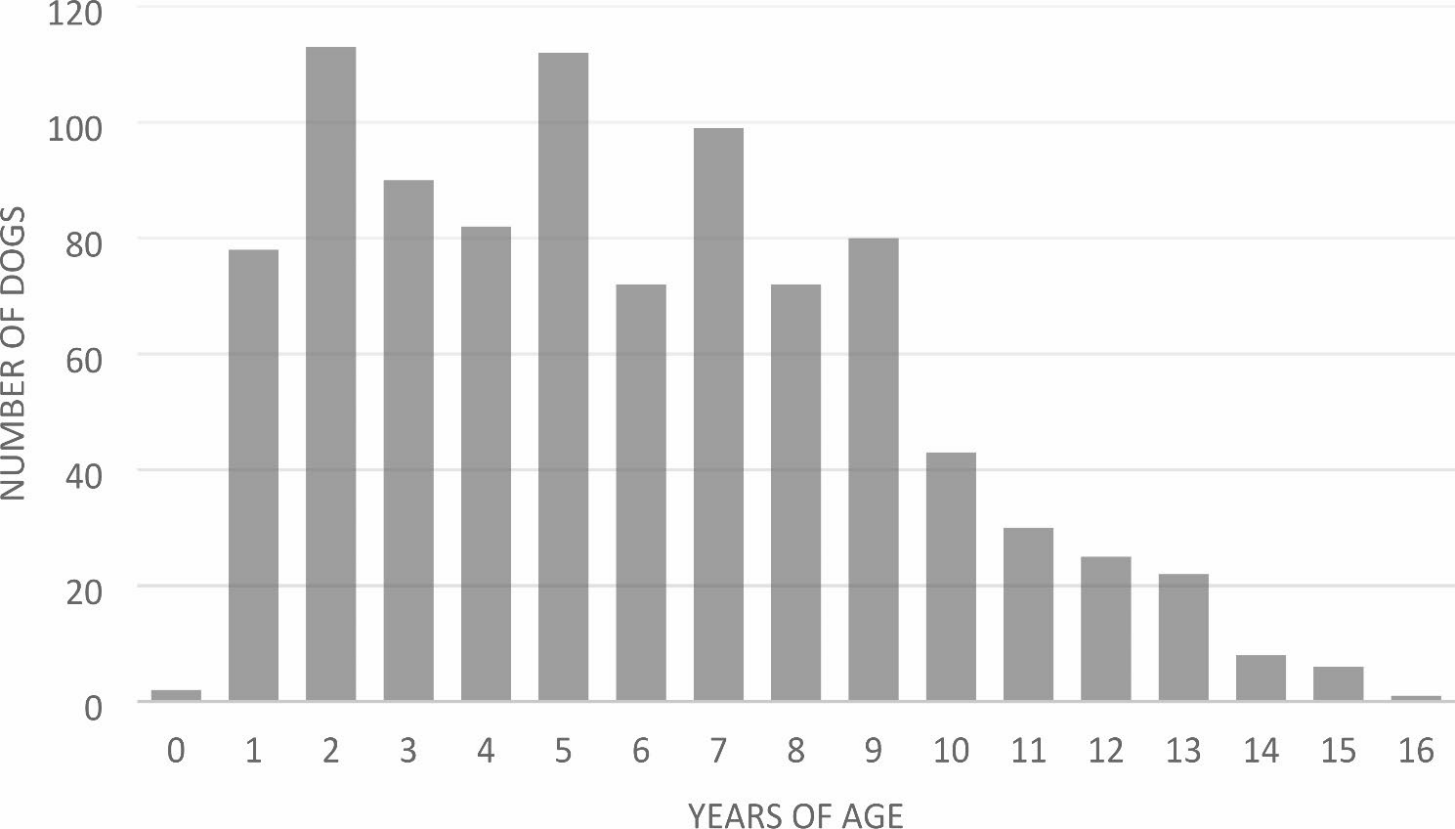
Distribution of age of living dogs (*n* = 935). Legend: Distribution of age in years, of living dogs included in the survey (*n* = 935)

The age of the living dogs spanned from 2 months to 16 years (median 5, IQR 3–8 years). Age distribution is shown in Fig. 1.

### IMRD

Of all of the 935 living individuals, 28 dogs (3%) were reported as diagnosed with IMRD, of which 18 were males (64%) and ten (36%) were females, the difference between sexes was not statistically significant (*P* = 0.13). The median age of onset of disease was five years (IQR 2–6 years) with 75% developing disease before seven years of age. Owners reported the clinical signs of the dog. Several owners reported multiple clinical signs. Reported clinical signs are listed in Table 1. In total 13 respondents (1.4%) did not know if their dogs ever had a diagnosis of IMRD by their veterinarian. When going through their responses it was likely that at least three of these dogs had had IMRD, those dogs are, however, not included in the statistics of dogs with IMRD.

**Table 1:** Reported clinical signs of dogs diagnosed with IMRD (*n* = 28) included in the survey

*Clinical sign*	*Number of dogs*	*%*
Limping	16	57
Stiff gait	14	50
Signs of pain	13	46
Fever	5	18
Depression	4	14
No answer, unclear answer	2	7

Number of dogs having the specific clinical sign and the percentage of all dogs with IMRD with the particular sign

Of the total 28 dogs with IMRD, four were reported as positive for antinuclear antibodies (ANA) (14%) and nine were reported as negative (32%) by the owners. For ten of the dogs the respondent’s could not remember the result of the IIF-ANA test and another five dogs were never tested. The reported treatments for dogs with IMRD are shown in Table 2. The primary treatment was glucocorticoids and the adjuvant treatments used were cyclosporin, azathioprine, mycophenolate mofetil and tramadol.

**Table 2:** Treatments reported for dogs diagnosed with IMRD in the survey

Treatment	*Number of dogs*	%
Corticosteroids	20	71
NSAID	6	21
Antibiotics	1	4
None	1	4
Corticosteroids + adjuvant treatment	6	21
Total	28	100

Treatments reported for dogs diagnosed with IMRD (*n* = 28) in the survey in numbers and percentage. NSAID: non-steroid anti-inflammatory drugs

Of the total 28 dogs with IMRD, 15 dogs (54%) relapsed after the initial episode, eight (29%) of these dogs relapsed while still on treatment and the other seven (25%) after discontinuing of treatment. Four owners (14%) did not know or did not remember if their dog had relapsed. For the dogs that relapsed after discontinuing the treatment, three relapsed within one month, two after 1–2 months and two dogs relapsed more than six months after discontinuing the treatment. None of the living dogs reported to have IMRD were reported to be diagnosed with lymphoma.

### SRMA

Out of the 935 living individuals, 33 dogs (3.5%) of the included dogs, were reported as diagnosed with SMRA, of which 20 were males (61%) and 13 (39%) were females, the difference was not statistically significant (*P* = 0.54). One of the dogs with SRMA was also reported to have been diagnosed with IMRD later in life and therefore included in both groups. Of the 33 diseased dogs, 70% developed disease before 18 months of age. Reported clinical signs are listed in Table 3.

**Table 3:** Reported clinical signs of dogs diagnosed with SRMA (*n* = 33)

Clinical sign	*Number of dogs*	%
Signs of pain	18	55
Stiff gait	20	61
Fever	16	49
Depression	19	58
No answer, unclear answer	0	0

Number of dogs having the specific sign and the percentage of all dogs with SRMA with the particular sign

One of the dogs reported to have SRMA, probably suffers from IMRD. The clinical signs and age at diagnosis were in agreement with IMRD rather than for SRMA, but since the owner reported the dog to have been diagnosed with SRMA by a veterinarian the dog was included in the SRMA group. All dogs with SRMA were treated with corticosteroids and two respondents reported that their dog also had received adjuvant immunosuppressive treatment with azathioprine or cyclosporin A, respectively. Half of all dogs with SRMA (16/33, 48%) had relapse of disease. Twelve of these dogs (75%) relapsed after treatment was discontinued and four (25%) relapsed while still on treatment. For the twelve dogs that relapsed after treatment was discontinued, four relapsed within a month and six dogs relapsed more than six months after treatment was discontinued. Two of the dogs relapsed within two to six months after the end of the treatment. The two dogs receiving adjuvant immunosuppressant treatment both reported to relapse to disease. The data does not tell if adjuvant was used due to relapse or due to another reason. Both dogs relapsed while still on medication.

### Neoplasia

Of the 935 dogs, 129 (14%) were reported to have or have had a neoplasia of any kind. Four respondents (0.4%) did not know whether their dog had had a neoplasia. The median age for developing a neoplasia was 6 years (IQR 5–8 years) and thirty-eight (29%) of the dogs were reported to have more than one neoplasia. Of the 129 dogs, 29 dogs (22%) were reported to have mammary tumors, 19 had mast cell tumors (15%) and five (4%) had melanomas. In 58 of the cases (45%), the owner could not specify what type of neoplasia the dog had. Regarding treatment, the respondents most commonly reported surgery (74%). Other therapeutic interventions reported were chemotherapy (*n* = 4), radiation therapy (*n* = 1) and medical treatment with, for example, prednisolone (*n* = 2). Among the 129 dogs, the owners of 30 dogs (23%) reported the neoplasia had not been treated.

### Deceased dogs

In total, 177 individual dogs in the survey were not alive when the owner responded to the survey. The average age at death was 9.6 years (range 0–17), for one dog the year of death was wrongly stated and could not be corrected, that dog is not included in the analysis of age. There were 90 males, of which 29 were castrated (32%), and 87 females, of which 17 were neutered (20%). For six males and five females, the owners did not report information about neutering or castration status. The most common cause of death/euthanasia was neoplasia, almost 40% of the owners gave neoplasia or suspicion of it as the cause of death/euthanasia for their dog. See Table 4 for results.

**Table 4:** Causes of death/euthanasia (*n* = 177)

Reason for death/euthanasia	*Number of dogs*	%
High age	18	10.2
Neoplasia or suspicion of neoplasia	68	38.4
immune mediated disease	16	9.0
Kidney or liver disease	11	6.2
Heart disease	5	2.8
Neurological disease or musculoskeletal disease	29	16.4
Behavior	7	4.0
Unknown	23	13.0
*Total*	*177*	*100*

Reasons for death of dogs included in the survey as stated by the respondent. Answers in numbers and percentage

Lymphoma was reported as cause of death for six dogs (3.4%); none of those were reported to have been diagnosed with either SRMA or IMRD. For seven dogs (4%), neoplasia in the spleen or rupture of the spleen due to neoplasia was reported as the cause of death/euthanasia.

## Discussion

In this study, we present results from a questionnaire to owners of Nova Scotia Duck Tolling Retrievers (NSDTRs) in Sweden, regarding their dogs’ health. We received responses regarding a large number of individual dogs, which constitutes 20% of the population of NSDTRs in Sweden. The response counts over different ages and sex corresponds to the typical demographic profile of the general dog population, i.e. most responses were obtained from comparably young dogs and fewer from the older dogs. This study reports an estimated lifetime prevalence of immune mediated rheumatic disease (IMRD) and steroid-responsive meningitis-arteritis (SMRA) in the studied population of Swedish NSDTRs of 3.0 and 3.5% respectively. Of the living dogs, 14% were reported to have a neoplasia of some kind and almost 40% of the deceased dogs were euthanized due to neoplasia or suspicion of neoplasia.

The prevalence of IMRD among the living dogs in this study (3.0%) corresponds well to a previous study based on insurance data where 0.35% of the NSDTRs had an IMRD diagnosis and 3.3% had an “IMRD possible”, together these groups constitute 3.7% [3].

Systemic lupus erythematosus (SLE) in humans is a disease with a significantly higher prevalence in women [14]. One previous study of IMRD in NSDTRs did not reveal any skewed sex distribution, while another showed a higher incidence of IMRD among female NSDTRs [3, 4]. In this survey, there were more males with IMRD than females; however, the number of individual dogs with IMRD was low, and the disparity between sexes did not reach statistical significance. We continue to believe that there is no preference for either sex in terms of IMRD occurrence in NSDTRs. The age for developing disease is in agreement of a previous study, as are the clinical signs reported by the owners [4]. Limping, stiff gait and pain were the most common complaints as expected with a disease that causes arthritis. Thirteen owners responded that they did not know if their dog suffered from IMRD. At least some of these cases were likely to have IMRD based on the clinical signs reported by the owner. Establishing a diagnosis of IMRD can be challenging for the veterinarian and this might be one of the reasons for the owners not knowing whether their dog is affected by the diseases or not. Clinical examination, neurological examination, x-rays and IIF-ANA test are all examples of what is often included in a work up for these dogs, however, the presentation of the individual dog as well as the financial situation of the owner and the diagnostic equipment of the clinic visited together determine what or which diagnostic procedures that are undertaken [15]. There was a smaller percentage of dogs with IMRD that was positive for ANA in this survey (14%) than reported by others, (70%), where ANA reactivity was measured in contrast to our study where we asked about what owners remember [4]. Ten of 28 owners reported that they did not recall whether their dog’s serum had been analyzed for ANAs, complicating the interpretation of ANA reactivity.

The treatments reported for IMRD is, as expected, dominated by corticosteroids, which is the treatment of choice [7]. Some dogs can be managed by NSAID alone [7]. The authors’ experience is that the less severe cases sometimes respond well to treatment with NSAID, but more severely affected cases generally need immunosuppressive treatment with corticosteroids or other immunomodulatory drugs. For the IMRD cases reported in the survey, three of the owners reported that their dogs were first put on NSAID but were switched over to glucocorticoids due to lack of response.

Adjuvant treatment is used for dogs with autoimmune disease when the response to corticosteroids is suboptimal or when side effects are too severe [7]. Among the dogs with IMRD, over 20% were treated with adjuvant, the data does not tell whether this was due to failure of response to corticosteroids or to side effects of the corticosteroid treatment. There is only one publication that has evaluated response to corticosteroid treatment in NSDTRs with IMRD, which concluded that 16 of the 25 dogs responded well to corticosteroids [4]. There are to this date no studies regarding how common it is for dogs with IMRD to need adjuvant treatment.

More than half of the dogs with a diagnosis of IMRD were reported to have relapse of disease after initial event. This is expected due to the normally episodic nature of the disease. However, relapse of disease can also be due to failure of treatment, i.e. starting doses of glucocorticoids are too low or period of treatment is too short [7]. It is important to customize the treatment to every patient and their clinical signs as well as the response to treatment and possible adverse side effects of the glucocorticoids. Since IMRD is a chronic disease, failure of treatment significantly reduces animal welfare, and it is therefore important to continue to deepen the knowledge about this disease through studies.

The estimated lifetime prevalence for SRMA in the studied population was 3.5%. There are no previous reports on the prevalence of SRMA among Swedish NSDTRs. A study from 2015 reported an incidence of SRMA in Swedish NSDTRs to be 20 per 10 000-dog years at risk (DYAR) when studying insurance data [3]. That gave NSDTRs 12-times higher risk of developing SRMA compared to all other breeds. A study from 2008 estimated the prevalence of SRMA in Norwegian NSDTRs to be 2.5% [2]. In an other study the prevalence of SRMA in all breeds was estimated to be 0.6% of all cases admitted to the small animal hospital of Glasgow University during the period of May 2006 and May 2008 [16].

In this survey, half of all dogs with SRMA were reported to have relapse of disease. Several previous studies have reported a high relapse rate of SRMA [11, 17]. In a short communication from 2013, relapse was reported in more than half of included NSDTRs [5]. That was a larger proportion of relapse than reported by another study of Norwegian NSDTRs with SRMA where 33.3% relapsed after withdrawal of therapy [2]. Corticosteroids are considered the go-to treatment but there is no treatment protocol known for being the most efficient [13]. Despite a high relapse rate, SRMA is considered to have a fair to good prognosis for complete recovery [13]. However, the present and previous studies, highlight the fact that owners should be informed of the high risk for relapse in disease [2, 5, 11, 17].

Almost 14% of the living dogs had or had had a neoplasia of any kind. The type of neoplasia was often not known by the owner, but mammary tumors and mast cell tumors were the most commonly reported. Mast cell tumors are common skin tumors in dogs, and different studies estimates the prevalence to 7–21% of all skin tumors [18, 19]. Other retriever breeds, such as Labrador retrievers and Golden retrievers have shown to be at increased risk for developing mast cell tumors [20].

When investigating the group of dead dogs, we divided them into eight different categories depending on the reason of death. The largest group included dogs that died or were euthanized due to neoplasia, almost 40% of the owners stated neoplasia or suspicion of it as the main reason for death/euthanasia. This is a larger proportion of death due to neoplasia than reported from other studies [21–23]. In a study from 2010 a mortality rate of 27% due to cancer was reported and in another study cancer was the cause of death in 14.5% of the cases [21, 22]. In a study of mortality in Swedish dogs of different breeds, 18% died because of neoplasia [23]. Although mortality rates due to neoplasia varies significantly between breeds, it still stands as the most common reason for death, even in the breeds with lower risk [24]. Still, 40% mortality due to suspected or confirmed neoplasia is high, but close to earlier reported mortality figures for e.g. Bernese Mountain Dog, Golden Retriever, Scottish Terrier, Bouvier des Flandres and Boxer, spanning between 44 and 55% cancer related mortality [24]. Research conducted on humans indicates that individuals with dysregulated immune responses and immune-mediated diseases are at a higher risk of developing neoplasia [25, 26]. This could be one reason for the high neoplasia-related mortality among the NSDTRs but other studies are needed to investigate this matter. The data in our survey, with both a fairly early age for developing neoplasia, together with a mortality rate close to the top ranked breeds in other studies, calls for a higher vigilance amongst veterinarians to early consider neoplasia as a differential diagnosis for NSDTRs. Early detection of neoplasia enables higher rate of successful treatments and often with a smaller impact on quality of life for the individual dog. Raising the awareness of both autoimmune disease and neoplasia in the NSDTRs may lead to increased animal welfare and potentially enable measures to reduce the prevalence of these diseases in the breed.

Prevalence data is always challenging to establish. In diseases like IMRD where clinical signs vary and there is no gold standard for establishing the diagnosis, estimating a prevalence is even harder. There is a lack of strict inclusion criteria for IMRD [27]. For humans there is a list of inclusion criteria for diagnosing SLE. Despite this, diagnosing SLE in humans is not always easy due to the many variants of the disease [28]. In this study, we investigated the health and disease of NSDTRs as reported by dog owners. It does not include data from medical records. Medical records would give more information about the dog’s health and illness and give details that owners might not remember. There is also a gap between information given by the veterinarian and what the owner remembers, especially if time has passed since information was given, known as recall bias. This could give an insecurity regarding if the diagnosis is correct. Nevertheless, both IMRD and SRMA typically manifest in noticeable clinical signs, making it probable that owners would remember and report such occurrences. In addition, by using owners as the source of information we were able to collect answers from a large part of the NSDTR population, something that would not have been possible if reviewing medical records since there is no common national register for animal health care medical records in Sweden. The prevalence of disease in this sample could be a slight over estimation since owners with diseased dogs are more likely to answer a survey. It should be noted that this is an estimation and that an exact prevalence is unlikely to ever be established.

## Conclusion

This study reports an estimated lifetime prevalence of IMRD and SMRA in Swedish NSDTRs to be 3.0 and 3.5% respectively. In this study, 14% of the living dogs were reported to have a neoplasia of some kind and almost 40% of the deceased dogs were euthanized due to neoplasia or suspicion of it. This is something to be aware of, both as a veterinarian and as dog owner and highlights the need for further investigations on the mechanisms behind immune mediated diseases and neoplasia in the breed.

## Electronic supplementary material

Below is the link to the electronic supplementary material.

Supplementary Material 1: Questionnaire distributed to owners of Nova Scotia Duck Tolling Retrievers in Sweden.

## Data Availability

The data is available from the authors upon reasonable request. The data are not publicly available due to them containing information that could compromise research participant privacy.

## Abbreviations

ANA: Antinuclear antibodies
IMRD: Immune mediated rheumatic disease
IQR: Inter quartile range
NSAID: Non steroid anti-inflammatory drugs
NSDTR: Nova Scotia Duck Tolling Retriever
SKK: Swedish Kennel Club
SJV: Swedish board of agriculture
SLE: Systemic lupus erythematosus
SRMA: Steroid-responsive meningitis-arteritis
