# Supplementary material for: In sickness and health - a questionnaire based study regarding immune mediated diseases and neoplasia in Swedish Nova Scotia Duck Tolling Retrievers
Source: Acta Vet Scand. 2024 Aug 15;66:39. doi: 10.1186/s13028-024-00761-x (PMC11325609; doi:10.1186/s13028-024-00761-x)
Supplement: Supplementary file 1 — Supplementary Material 1: Questionnaire distributed to owners of Nova Scotia Duck Tolling Retrievers in Sweden. [file 13028_2024_761_MOESM1_ESM.pdf]

Welcome to the questionnaire regarding health and disease among Nova Scotia Duck Tolling Retriever 2022!

The purpose of the survey is to gather data, to collect data to map the health status of the Swedish population of Nova Scotia Duck Tolling Retrievers. Through the mapping, we can get information about how common various diseases are or whether any disease is increasing within the breed. That knowledge helps to lay a foundation for breeding recommendations, which can lead to healthier dogs being bred. By participating and answering the questions about your dog, you can help us get as comprehensive a picture as possible.

Participation in the survey is of course voluntary, but we hope you will participate, as your answers cannot be replaced by someone else's.

By participating in the survey, you agree that the data in the survey may be used by researchers for the purpose of investigating the health status of the breed. The legal basis for personal data processing is public interest. Individual respondents may be contacted for further questions for research purposes. Collected information will, in such a way that it cannot be derived to an individual dog, be shared with the Breed club in order to produce statistics for the health status of the breed, which will be published in the club newspaper and on the breed club's homepage. Data collected via the survey will be saved for the duration of the research project. As a state authority, SLU is subject to the principle of openness. You can withdraw your consent at any time. No information about you or your dog will then be saved. You can also withdraw your consent at any time by contacting us. Your data will then not be used.

The Swedish University of Agriculture (SLU) is responsible for the processing of your personal data. If you have comments on SLU's processing of personal data, you can contact SLU's data protection officer via dataskydd@slu.se or 018-67 20 90. If you are not satisfied with SLU's answer, you can contact SLU with a complaints regarding processing of your personal data to the Swedish Privacy Protection Agency (IMY), imy@imy.se or telephone 08-657 61 00

You are answering the survey for an individual dog. If you have several dogs, you can answer several times. You can also answer for dogs that are no longer alive.

The questionnaire will be open until 30th of june 2022.

If you have questions you are welcome to contact:

Malin Nilsson, leg. vet. PhD student
malin.nilsson@slu.se 018-671354

Henrik Rönnberg, leg. vet. VMD, diplomate ECVIM-CA (onc) professor
henrik.ronnberg@slu.se

Helene Hamlin, leg. vet. VMD, docent
helene.hamlin@slu.se

Page 1

General information. At first, there will be general questions regarding your dog. This questionnaire is about one individual dog. If you have more than one dog, you can answer the questionnaire one time for every individual dog.

The registered name of the dog:

What do you call your dog?

The dogs registration number:

Date of birth: (YYMMDD)

Page 2

Is the dog alive?

Yes

No

Page 3

What year did the dog die or was euthanized?

Page 4

What was the cause of death or euthanasia? You are very welcome to answer the rest of the questionnaire even though the dog is dead.

Page 5

The home of the dog

Does the dog live with you most of the time?

Yes

No

Don´t know

Page 6

The dogs sex

What is the sex of the dog?

Female

Neutered female

Male

Castrated male

Page 7

Castration

Age at castration/neutering:

Page 8

Castration

Cause of castration/neutering?

Page 9

Testicles

Does your dog have both testicles in the scrotum?

Yes

No, only one of them

No, none of them

Don’t know

Page 10

Testicles at delivery

Did your dog have its testicles in the scrotum at age of delivery (8-9 weeks)?

Yes, both

Yes, but only one of them

No, none of them

Don’t know/cant remember

Page 11

Umbilical hernia

Does your dog have or had had an umbilical hernia?

No

Yes

Don´t know

Page 12

Umbilical hernia

How large would you estimate the hernia to be/was?

Page 13

Umbilical hernia

Have your dog had surgery for its umbilical hernia?

No

Yes

Don´t know/cant remember

Page 14

Perineal hernia

Does your dog have or had had an perineal hernia?

No

Yes

Don´t know

Page 15

Perineal hernia

Have your dog had surgery for its perineal hernia?

No

Yes

Don´t know

Page 16

Inguinal hernia

Does your dog have or had had an inguinal hernia?

No

Yes

Don´t know

Page 17

Inguinal hernia

Have your dog had surgery for its perineal hernia?

No

Yes

Don´t know

Page 18

Teeth

Does your dog have normal dentation? (Permanent teeth: in total 20 teeth in upper jaw and 22 in lower jaw)

Yes

No

Don´t know

Page 19

Dental occlusion

Does your dog have a normal occlusion?

Yes

No

Don´t know

Page 20

Dental occlusion

What kind of malocclusion did/does your dog have?

Over bite

Prognathism

Wry bit

Don’t know

Other

Page 21

Diseases – “toller disease”

Now we are going to ask a number of questions regarding different diseases. We will start with toller disease. Toller disease is the common name for the two diseases of meningitis/SRMA and immune mediated rheumatic disease/rheumatic disease/IMRD/SLE-like disease. These two diseases can be named one of their many names or as toller disease. In this questionnaire, they will be named SRMA and IMRD and not toller disease.

Have your dog ever, by a veterinarian, been diagnosed with meningitis/SRMA?

No

Yes

Don´t know

Page 22

Diseases – SRMA/meningitis

How old was the dog when the disease began?

Page 23

Diseases – SRMA/meningitis

What clinical signs did the dog have?

Page 24

Diseases – SRMA/meningitis

How was the diagnosis stated? Common interventions are clinical exam, blood samples, spinal fluid sample, imagine diagnosis like computed tomography.

Page 25

Diseases – SRMA/meningitis

If the dog was tested for ANA, what was the result of the test?

Positive (ANA found)

Negative (ANA not found)

Was not tested

Don't know

Page 26

Diseases – SRMA/meningitis

What kind of treatment was given to the dog? Please, specify both medication, dose and length of treatment if you remember. Often used medications are different types of glucocorticoids like prednisolone or Medrol, different NSAIDs (anti-inflammatory and pain-relieving drug, like metacam, rimadyl or onsior) or other immunomodulatory medications like Atopica or Imurel.

Page 27

Diseases – SRMA/meningitis

Have the dog relapsed in disease?

Yes, during treatment.

Yes, after discontinuing treatment.

No

Don't know

Page 28

Diseases – SRMA/meningitis

What dose of medication was the dog on at time for relapse? If you remember, please specify how long the dog had been on the same dose before relapse.

Page 29

Diseases – SRMA/meningitis

How long after discontinuing of medication, did the dog relapse?

Page 30

Diseases - IMRD/arthritis/SLE-like disease

Have your dog ever, by a veterinarian, been diagnosed with IMRD/arthritis/SLE-like disease?

No

Yes

Don’t know

Page 31

Diseases - IMRD/arthritis/SLE-like disease

How old was the dog when the disease began?

Page 32

Diseases - IMRD/arthritis/SLE-like disease

What clinical signs did the dog have?

Page 33

Diseases - IMRD/arthritis/SLE-like disease

How was the diagnosis stated? Common interventions are clinical exam, blood samples, x-ray of joints, sampling of joint fluid, testing for ANA etc.

PAGE 34

Diseases - IMRD/arthritis/SLE-like disease

If the dog was tested for ANA, what was the result of the test?

Positive (ANA found)

Negative (ANA not found)

Was not tested

Don’t know

PAGE 35

Diseases - IMRD/arthritis/SLE-like disease

What kind of treatment was given to the dog? Please, specify both medication, dose and length of treatment if you remember. Often used medications are different types of glucocorticoids like prednisolone or Medrol, different NSAIDs (anti-inflammatory and pain-relieving drug, like metacam, rimadyl or onsior) or other immunomodulatory medications like Atopica or Imurel.

PAGE 36

Diseases - IMRD/arthritis/SLE-like disease

Have the dog relapsed in disease?

Yes, during treatment.

Yes, after discontinuing treatment.

No

Don’t know

PAGE 37

Diseases - IMRD/arthritis/SLE-like disease

What dose of medication was the dog on at time for relapse? If you remember, please specify how long the dog had been on the same dose before relapse.

PAGE 38

Diseases - IMRD/arthritis/SLE-like disease

How long after discontinuing of medication, did the dog relapse?

PAGE 39

Diseases – other immune mediated diseases

Have your dog ever, by a veterinarian, been diagnosed with another immune mediated disease like IMHA (immune mediated haemolytic anaemia), DLE (discoid lupus erythematosus) or IMHA (immune mediated haemolytic anaemia)?

No

Yes

Don’t know

PAGE 40

Diseases – other immune mediated diseases

What kind of immune mediated disease?

PAGE 41

Diseases – other immune mediated diseases

How old was the dog when the disease developed?

PAGE 42

Diseases – other immune mediated diseases

What clinical signs did the dog have?

PAGE 43

Diseases – other immune mediated diseases

If the dog was tested for ANA, what was the result of the test?

Positive (ANA found)

Negative (ANA not found)

Was not tested

Don’t know

PAGE 44

Diseases – other immune mediated diseases

What kind of treatment did the dog receive?

PAGE 45

Diseases – lameness

Have your dog ever had problems with lameness?

No

Yes

Don’t know

PAGE 46

Diseases – lameness

Was the lameness examined by a veterinarian?

No

Yes

Don’t know

PAGE 47

Diseases – lameness

What was the cause of the lameness?

PAGE 48

Diseases – lameness

What clinical signs did the dog have?

PAGE 49

Diseases – lameness

How old was the dog when it developed the lameness?

PAGE 50

Diseases – lameness

If the dog was tested for ANA, what was the result of the test?

Positive (ANA found)

Negative (ANA not found)

Was not tested

Don't know

PAGE 51

Diseases – lameness

What treatment did/does the dog receive?

PAGE 52

Diseases – lameness

Has the lameness reoccurred?

No

Yes

Don't know

PAGE 53

Diseases - lymphedema

Have your dog ever, by a veterinarian, been diagnosed with lymphedema or another problem with the lymphatic system resulting in severely swollen limbs?

No

Yes

Don't know

PAGE 54

Diseases - lymphedema

What clinical signs did the dog have?

PAGE 55

Diseases - lymphedema

How old was the dog when the problems developed?

PAGE 56

Diseases - lymphedema

If the dog was tested for ANA, what was the result of the test?

Positive (ANA found)

Negative (ANA not found)

Was not tested

Don't know

PAGE 57

Diseases - lymphedema

What treatment did/does the dog receive?

PAGE 58

Diseases – thyroid gland

Have your dog ever, by a veterinarian, been diagnosed with hypothyroidism?

No

Yes

Don't know

PAGE 59

Diseases – thyroid gland

How old was the dog when the disease developed?

PAGE 60

Diseases – thyroid gland

What clinical signs did the dog show?

PAGE 61

Diseases – thyroid gland

What treatment did/does the dog receive?

PAGE 62

Diseases – Addison's disease

Is your dog tested for juvenile Addison's disease and if so what was the result of the test?

Yes, tested as normal/clear

Yes, tested as carrier

Yes, tested as affected

No, but clear by parents

No, not tested

Don't know

PAGE 63

Diseases – Addison's disease

Have your dog ever, by a veterinarian, been diagnosed with Addison's disease or juvenile Addison's disease)

No

Yes

Don’t know

PAGE 64

Diseases – Addison’s disease

How old was the dog when the disease developed?

PAGE 65

Diseases – Addison’s disease

What kind of clinical signs did the dog have?

PAGE 66

Diseases – Addison’s disease

What treatment did/does the dog receive?

PAGE 67

Diseases - endocrine

Have your dog ever, by a veterinarian, been diagnosed with another endocrine disease like diabetes mellitus or Cushing’s syndrome?

No

Yes

Don’t know

PAGE 68

Diseases - endocrine

Which disease?

PAGE 69

Diseases - endocrine

How old was the dog when the disease developed?

PAGE 70

Diseases - endocrine

What clinical signs did the dog have?

PAGE 71

Diseases - endocrine

What treatment did/does the dog receive?

PAGE 72

Diseases – Tumors

Now we are going to as questions related to tumors or neoplasia's. These can be both benign or malignant. We count all types of neoplasia's/lumps/tumors/cancers.

Have your dog ever, by a veterinarian, been diagnosed with neoplasia's/lumps/tumors/cancers anywhere on or in the body?

No

Yes

Don't know

PAGE 73

Diseases – Tumors

What kind of neoplasia/lump/tumor/cancer? Please specify the tumor type if you can. It you cant, please state where the tumor was located (for instance in the skin).

PAGE 74

Diseases – Tumors

How old was the dog when it the neoplasia/lump/tumor/cancer was discovered?

PAGE 75

Diseases – Tumors

How was the dog treated? Multiple choices can be made.

No treatment

Surgery

Chemotherapy

Radiation

Immunotherapy

Other

PAGE 76

Diseases – Tumors

After treatment, have the neoplasia/lump/tumor/cancer reoccurred?

No

Yes

Don't know

PAGE 77

Diseases - Allergy

Have your dog ever, by a veterinarian, been diagnosed with an allergy?

No

Yes

Don’t know

PAGE 78

Diseases - Allergy

What kind of allergy?

How does the allergy show?

PAGE 79

Diseases - Allergy

How old was the dog when the allergy was discovered?

PAGE 80

Diseases – Allergy

What treatment did/does the dog receive?

Treatment can be different kind of feed, mediations like glucocorticoids, shampoos or different ointments.

PAGE 81

Diseases - Eyes

Have your dog ever, by a veterinarian, been diagnosed with conjunctivitis or other diseases/problems related to the eyes?

No

Yes

Don’t know

PAGE 82

Diseases - Eyes

Which kind of disease/problems?

PAGE 83

Diseases - Eyes

How old was the dog when the disease/problem developed?

PAGE 84

Diseases - Ears

Have your dog ever, by a veterinarian, been diagnosed with diseases of the ears?

No

Yes

Don’t know

PAGE 85

Diseases - Ears

What kind of ear disease?

PAGE 86

Diseases - Ears

How old was the dog when the ear disease developed?

PAGE 87

Diseases – Skin

Have your dog ever, by a veterinarian, been diagnosed with some kind of skin disease?

No

Yes

Don’t know

PAGE 88

Diseases – Skin

What kind of skin disease?

PAGE 89

Diseases – Skin

How old was the dog when the skin disease developed?

PAGE 90

Diseases – Skin

What treatment did/does the dog receive?

PAGE 91

Diseases – Gastrointestinal tract

Have your dog ever had recurrent problems related to the gastrointestinal tract like diarrhoeas, vomiting or sensitivity against different feed?

No

Yes

Don’t know

PAGE 92

Diseases – Gastrointestinal tract

What kind of problems? Multiple choices can be made.

Vomiting

Regurgitation

Nausea

Diarrhoea

Constipation

Blood in stools

Mucus in the stools

Inappetence

Other

PAGE 93

Diseases – Gastrointestinal tract

Have your dog ever been through veterinary examinations due to its problems with the gastrointestinal tract?

No

Yes

Don’t know

PAGE 94

Diseases – Gastrointestinal tract

Which diagnosis have the dog received?

PAGE 95

Diseases – Gastrointestinal tract

How old was the dog when the problems with the gastrointestinal tract developed?

PAGE 96

Diseases – Gastrointestinal tract

What kind of treatment have the dog received for its problems with gastrointestinal tract? Treatment can be different kinds of feed, medications like glucocorticoids or others.

PAGE 97

Diseases – Infections

Have you dog ever had an infection or inflammation that has led to the need for veterinary care?

No

Yes

Don’t know

PAGE 98

Diseases – Infections

What kind of infection or inflammation?

PAGE 99

Diseases – Infections

How old was the dog when the infection/inflammation occurred?

PAGE 100

Diseases - Heart

Have your dog ever, by a veterinarian, been diagnosed with med heart disease?

No

Yes

Don’t know

PAGE 101

Diseases - Heart

What kind of heart disease?

PAGE 102

Diseases - Heart

How old was the dog when the heart disease was discovered?

PAGE 103

Diseases – Seizures

Have your dog ever, by a veterinarian, been diagnosed with seizures or epilepsy?

No

Yes

Don’t know

PAGE 104

Diseases – Seizures

How old was when the seizures or epilepsy was diagnosed?

PAGE 105

Diseases – Seizures

How was the diagnosis set?

PAGE 106

Diseases – Seizures

Do your dog receive any treatment for its seizures or epilepsy?

No

Yes

Don’t know

PAGE 107

Diseases – Seizures

What kind of treatment? Please specify both type of medication and dose. Commonly used medications are fenemal, pheoleptil, epirepress, epityl, potassium bromide, keppra and others.

PAGE 108

Diseases – Skeletal/joint problems

Have your dog ever had any kind of skeletal and/or joint disease like chondrodysplasia, dwarfism, FCP (fragmented choronoid process), OCD (osteochondrosis) etc?

No

Yes

Don’t know

PAGE 109

Diseases – Skeletal/joint problems

What kind of problems?

PAGE 110

Diseases – Skeletal/joint problems

How old was the dog when the problems developed?

PAGE 111

Diseases – Skeletal/joint problems

Did/does your dog need any treatment because of this? What kind of treatment?

PAGE 112

Diseases – Urinary tract

Have your dog ever, by a veterinarian, been diagnosed with med kidney disease or disease of the urinary tract?

No

Yes

Don’t know

PAGE 113

Diseases – Urinary tract

What kind of disease?

PAGE 114

Diseases – Urinary tract

How old was the dog when the disease developed?

PAGE 115

Diseases – Urinary tract

Have the dog been needing any treatment for its disease of the urinary tract? What kind of treatment? Examples of treatment can be antibiotics, feed, hypotensive medications etc.

PAGE 116

Diseases - Liver

Have your dog ever, by a veterinarian, been diagnosed with liver disease?

No

Yes

Don’t know

PAGE 117

Diseases - Liver

What kind of liver disease?

PAGE 118

Diseases - Liver

How old was the dog when the liver disease developed?

PAGE 119

Diseases - Liver

Did the dog need any treatment?

No

Yes

Don’t know

PAGE 120

Diseases - Liver

What kind of treatment? Example of treatments can be different feed, ursodeoxycholic acid, glucocorticoids or gastro protective medications etc.

PAGE 121

Diseases - Liver

What kind of treatment? Example of treatments can be different feed, ursodeoxycholic acid, glucocorticoids or gastro protective medications etc.

PAGE 122

Diseases - prostate

Have your dog ever had problems related to prostate, like inflammation or enlarged prostate?

No

Yes

Don’t know

PAGE 123

Diseases - prostate

What kind of problem with the prostate?

PAGE 124

Troubles with heat

Have your dog experienced problems with heat like, long heats, disrupted heat etc?

No

Not applicable, have not had her first heat

Yes

Don’t know

PAGE 125

Troubles with heat

What kind of problems heat have your dog experienced?

PAGE 126

Troubles with mating

Have your dog ever had troubles mating?

No

Not applicable, have never been mated

Yes

Don’t know

PAGE 127

Trouble with mating

What kind of troubles related to mating?

PAGE 128

Troubles with pregnancy

Have your dog ever had problems related to pregnancy, as abortion, miscarriage, reabsorbed foetuses etc?

No

Not applicable, have never been pregnant

Yes

Don’t know

PAGE 129

Troubles with pregnancy

What kind of problems regarding pregnancy have your dog experienced?

PAGE 130

Diseases – uterine

Have your dog ever been diagnosed with a uterine infection (pyometra)?

No

Yes

Don’t know

PAGE 131

Diseases – uterine

How old was the dog when she developed uterine infection (pyometra)?

PAGE 132

Diseases – uterine

How was the uterine infection/pyometra treated?

Surgical by castration

With antibiotics and no surgery

Was not treated, healed anyway

Was not treated, the dog was euthanized

Don’t know

PAGE 133

Mentality

Has your dog got the mentality you expected?

Yes

No

Don’t know

PAGE 134

Mentality

Please describe what you regard as the problem?

PAGE 135

Other diseases

Have your dog ever, by a veterinarian, been diagnosed with any other disease not discussed in this questionnaire?

No

Yes

Don’t know

PAGE 136

Other diseases

Which disease?

PAGE 137

Other

Do you have any additional comments regarding your dog?

PAGE 138

Information – dog owner

Lastly, we as for you contact details.

Name dog owner:

Phone number dog owner:

E-mail address dog owner:

PAGE 139

THANK YOU!

Thank you very much for taking the time to answer all questions! The data in this questionnaire will be compiled and published at the Toller Breed Club webpage among others.
